# *AnnoSINE*: a short interspersed nuclear elements annotation tool for plant genomes

**DOI:** 10.1093/plphys/kiab524

**Published:** 2021-11-18

**Authors:** Yang Li, Ning Jiang, Yanni Sun

**Affiliations:** 1 Department of Electrical Engineering, City University of Hong Kong, Kowloon, Hong Kong SAR, China; 2 Department of Horticulture, Michigan State University, East Lansing, Michigan 48824, USA

## Abstract

Short interspersed nuclear elements (SINEs) are a widespread type of small transposable element (TE). With increasing evidence for their impact on gene function and genome evolution in plants, accurate genome-scale SINE annotation becomes a fundamental step for studying the regulatory roles of SINEs and their relationship with other components in the genomes. Despite the overall promising progress made in TE annotation, SINE annotation remains a major challenge. Unlike some other TEs, SINEs are short and heterogeneous, and they usually lack well-conserved sequence or structural features. Thus, current SINE annotation tools have either low sensitivity or high false discovery rates. Given the demand and challenges, we aimed to provide a more accurate and efficient SINE annotation tool for plant genomes. The pipeline starts with maximizing the pool of SINE candidates via profile hidden Markov model-based homology search and de novo SINE search using structural features. Then, it excludes the false positives by integrating all known features of SINEs and the features of other types of TEs that can often be misannotated as SINEs. As a result, the pipeline substantially improves the tradeoff between sensitivity and accuracy, with both values close to or over 90%. We tested our tool in *Arabidopsis thaliana* and rice (*Oryza sativa*), and the results show that our tool competes favorably against existing SINE annotation tools. The simplicity and effectiveness of this tool would potentially be useful for generating more accurate SINE annotations for other plant species. The pipeline is freely available at https://github.com/yangli557/AnnoSINE.

## Teaser

By integrating all known features of short interspersed nuclear elements (SINEs), AnnoSINE is a tool for both accurate and fast genome-scale SINE annotation in plant genomes.

## Introduction

Transposable elements (TEs), which are repetitive and mobile DNA sequences, are very abundant in most eukaryotic genomes. For example, TEs constitute ∼50% of the human genome (Cordaux and Batzer, 2009) and ˃80% of the maize (*Zea mays*) genome (Schnable et al., 2009). They are the largest component of the genetic material of most plants and play substantial roles in genome size variation (Zhao et al., 2016). Thus, TEs are a major source of genomic variability in plants.

TEs are mobile due to their capability to integrate into new positions in the genome. As TEs can be inserted into or near coding regions, their impact on gene composition, expression, and function has long been of interest (Lisch, 2013). There is accumulating evidence that TEs have regulatory roles (Seibt et al., 2016; Kögler et al., 2017). For example, Makarevitch et al. (2015) showed that some TEs may contribute to the response of nearby genes to abiotic stress in maize. The study of the stress-tolerant wild tomato species *Solanum pennellii* also suggested a complex interplay between TEs and stress-related genes (Bolger et al., 2014). Given the ubiquitousness of TEs in plant genomes and their complex interactions with genes, accurate TE annotation in genomes provides insightful information into understanding evolution, gene structure, and genome organization (Ou et al., 2019).

There are different types of TEs with different moving mechanisms and functions. TEs can be divided into two major groups based on whether they use RNA or DNA as the intermediate: Class I retrotransposons and Class II DNA transposons. Retrotransposons are DNA sequences that are first transcribed into an RNA intermediate and then reverse transcribed from RNA to DNA, employing a “copy-and-paste” transposition mechanism. In contrast, DNA transposons, which are not generated through an RNA intermediate, transpose through catalysis of transposase enzymes, using a “cut-and-paste” transposition mechanism (Finnegan, 1992; Kapitonov and Jurka, 2008). The Class I TEs can be further divided into two groups based on whether they have long terminal repeats (LTRs). LTR retrotransposons fall into the *Copia* and the *Gypsy* groups and are often responsible for genome size variation in plants (Kumar and Bennetzen, 1999). Non-LTR retrotransposons include long interspersed nuclear elements (LINEs) and short interspersed nuclear elements (SINEs; Panta et al., 2021). Different annotation tools have been developed for annotating all or some types of TEs. Among the different types of TEs, SINE annotation is particularly difficult. SINEs rely on an RNA intermediate in their copy-and-paste movement mechanism. They are small, highly heterogeneous, and possess degenerate sequence motifs or structural signatures (Wenke et al., 2011).

SINEs are not the most prevalent TEs in plants, despite the fact that some SINE elements could achieve very high copy numbers (Yoshioka et al., 1993). Although the percentages of genomic mass of SINEs in plant genomes are small (usually ˂1% of the genome), SINEs in plants have a profound impact on gene function and evolution (Deragon and Zhang, 2006; Baucom et al., 2009; Liu et al., 2009; Walters-Conte et al., 2011; Wenke et al., 2011; Seibt et al., 2016; Kögler et al., 2017). It is known that the presence of SINEs is associated with the modification of chromatin structure, which leads to the regulation of gene expression (Doğan and Liu, 2018) at different levels across the eukaryotic genome. In addition, SINEs can interact with transcription repressors and activators to weaken or enhance their functions (Ptashne, 2005). To better understand the organization, evolution, and diversity of SINE elements, it is important to develop a tool for genome-scale SINE annotation (Bennetzen, 2000).

**Figure 1:**
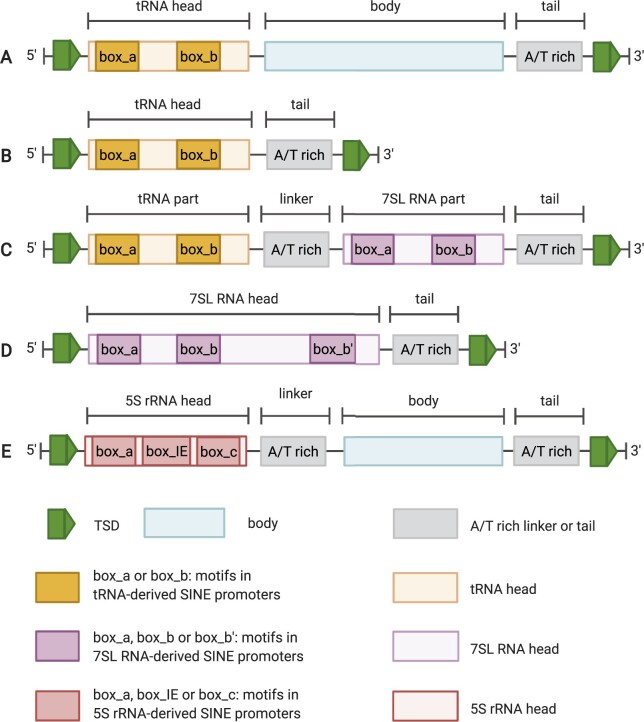
Typical SINE structures reproduced from Kramerov and Vassetzky (2011), Vassetzky and Kramerov (2013).

Compared to other transposons with well-conserved structures such as LTR retrotransposons, SINE annotation is still a major challenge. Although SINEs from different species have some common molecular structural features, these features are usually degenerate and are difficult to utilize for accurate SINE annotation in genomes. SINEs are short, often between 100 and 700 bp (Kovalchuk et al., 1998; Huang et al., 2012). The abundance of SINEs varies among genomes, so different species have different copy numbers. For example, the copy numbers of SINEs in *Arabidopsis thaliana*, rice (*Oryza sativa*), and pepper (*Capsicum annuum*) can differ greatly, from about 500 to 20,000 (Deragon and Zhang, 2006; Vassetzky and Kramerov, 2013). Some existing SINE annotation tools rely on the structural features of SINEs, which generally comprise three parts: head, body, and tail (Panta et al., 2021). The typical structures of SINEs are shown in Figure  1. Although the head regions of the 5′ end of most SINEs are derived from transfer RNAs (tRNAs) and some from 7SL RNA or 5S rRNA, not all SINEs show detectable homology with those ncRNAs. Some SINEs have two degenerated motifs named box A and B inside the head. However, neither their sequence conservation nor locations are prominent enough for accurate and sensitive SINE detection (Luan et al., 1993; Kapitonov and Jurka, 2008). The body region may be derived from a LINE. The tail region of the 3′-terminal has a variable length of simple repeats such as poly A, poly T, or other low complexity sequences (Luan et al., 1993). A better feature to use is the target site duplication (TSD) flanking the 5′- and 3′-termini, which are duplicated sequences ranging from 8 to 16  nucleotide (nt) and thus are identical upon insertion (Huang et al., 2012).

Among plants, sequence conservation of SINEs tends to be confined to related species, and the conservation often decays and disappears as the genetic distance increases. However, there are some exceptions. In Solanaceae, several families of SINEs are conserved among potato (*Solanum tuberosum*), tomato (*Solanum lycopersicum*), and *Nicotiana benthamiana*, which diverged 24 million years ago (Wenke et al., 2011; Särkinen et al., 2013), suggesting it is not rare for SINEs to be conserved among plants in the same family. One of the SINE families mentioned above, called Au, was detected from many monocot and dicot species, so this element likely originated prior to the divergence of monocots and dicots (Fawcett et al., 2006). Apparently, some SINEs are conserved among distantly related species, hence it is possible to identify those elements through homology-based approaches.

As demonstrated by Ou et al. (2019), the tradeoff between sensitivity and precision of SINE annotation by current tools is not satisfactory. SINE-Finder has the highest sensitivity of ∼60% among the three tested tools. Nevertheless, its accuracy can only reach 5%, suffering from a large number of false positives. In contrast, the most precise resource for SINE annotation, SINE Base, is associated with a sensitivity ˂50%.

In this study, we present a SINE annotation tool named *AnnoSINE* that carefully integrates multiple features of SINEs in order to achieve a better tradeoff between sensitivity and precision. Our experiments on cross-species SINE detection show that our tool competes favorably against existing SINE annotation tools.

## Results

**Figure 2:**
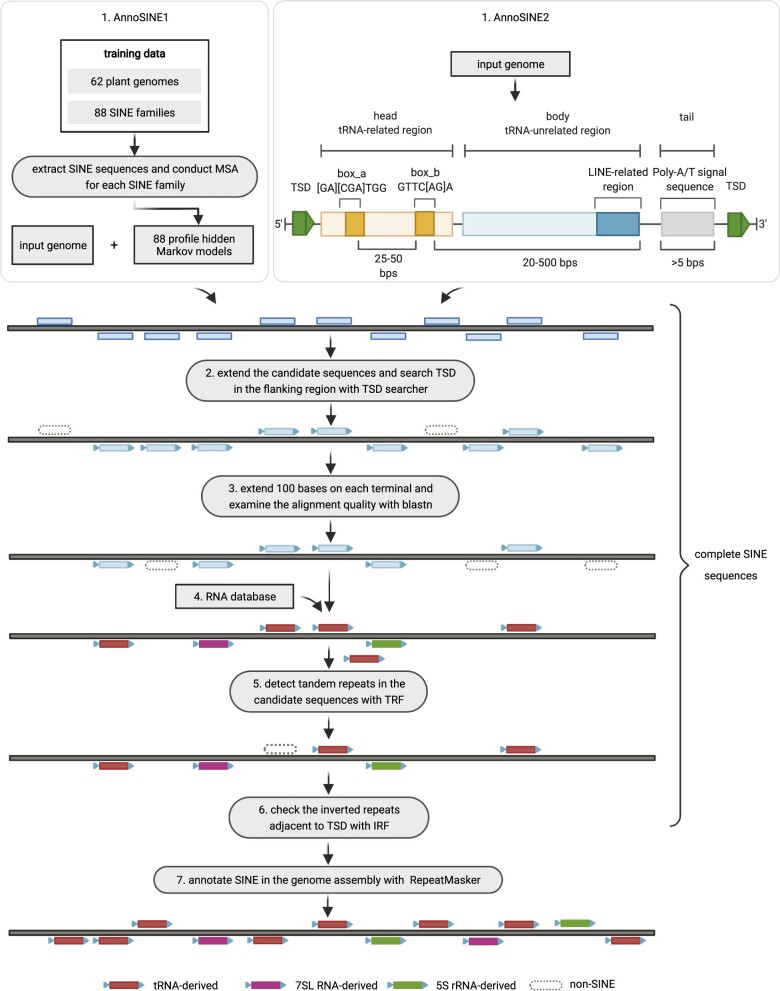
The pipeline of *AnnoSINE*. *AnnoSINE1*, and *AnnoSINE2* use different methods to generate initial SINE candidates. The first six steps aim to generate sequences of intact SINE elements with ˂80% similarity to each other (less redundancy between the SINEs). TSD Search tool: (Vassetzky and Kramerov, 2013); BLAST: (Eddy, 2011); Rfam, the ncRNA database (Kalvari et al., 2021); TRF, tandem repeat finder: (Benson, 1999); IRF (Warburton et al., 2004). The last step produces whole-genome SINE annotation.

The *AnnoSINE* pipeline is sketched in Figure  2. It has seven major components. The first one is to identify putative SINE candidates by applying hidden Markov model (HMM)-based homology search or structure-based de novo search. By allowing both strategies, the pipeline can recover as many SINE candidates as possible for input plants with different evolutionary distances to the training genomes. As this step can output false SINE candidates, the output will be subject to a series of filtering. In the second step, we search for TSD in the flanking region to further verify each SINE candidate. As TSD is an important feature of SINEs, this step is highly effective in eliminating non-SINEs. Although searching for TSD can be conducted in the later stage of the pipeline, removing false positives earlier would reduce the computation time of the downstream analysis. Third, we examine each candidate’s copy number and the copies’ multiple sequence alignment (MSA) in order to remove the sequences with low copy numbers or shifted/fragmented alignments. In the fourth step, we determine the superfamily of each candidate SINE sequence and remove candidates highly similar to known noncoding RNAs. In the fifth step, we exclude candidates containing a large proportion of tandem repeats. In the sixth step, we remove other DNA TEs by detecting inverted repeats adjacent to TSDs. These steps focused on identifying intact SINEs (i.e. seed sequences) in the query genome. Redundant seeds are filtered to generate the SINE library. After we obtain the nonredundant seed sequences, we apply RepeatMasker to generate genome-scale SINE annotation.

The input to *AnnoSINE* is the DNA sequence of a genome, and the output is the annotation (loci) of SINEs and libraries containing sequences of verified intact SINEs. In order to quantify the performance of *AnnoSINE*, we tested it in rice (*O.* *sativa)* and *A.* *thaliana* with the standard SINE library (see “Materials and methods”). When we tested our method, we used species masking, meaning that the training data (SINEs) do not contain any SINE from the test species. Thus, the cross-species SINE annotation experiments emulate SINE annotation in a newly assembled genome.

### Impact of BLAST-related parameters on the performance

**Figure 3:**
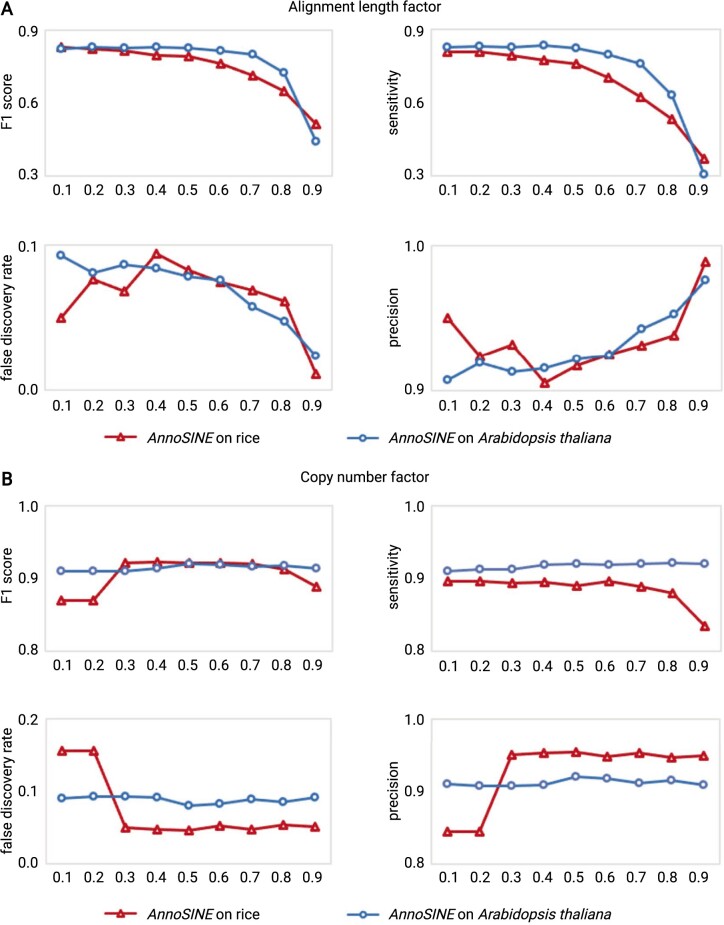
Impact of two factors on the SINE annotation performance. Red line: *AnnoSINE* for rice. Blue line: *AnnoSINE* for *A. thaliana*. A, The impact of the alignment length on the performance of SINE annotation. X-axis: “Alignment length factor” (α, defined in Section Materials and Methods.) B, The impact of copy number factor on the performance of SINE annotation. X-axis: “Copy number factor” (τ, defined in Section Materials and Methods).

In Step 3 of our method, we used BLAST to determine the copy numbers of each SINE. We applied the constraints on the alignment length (length factor α) and the minimum percentage of the copy number (copy number factor τ) to remove short, simple repeats and non-SINEs that fortuitously share sequence similarities with the SINE pHMMs or contained weak SINE structural features. Overall, with the increased stringency of the copy number factor, the sensitivity tends to decrease while the precision tends to increase. Some fluctuations could occur during the change of τ In conclusion, we recommend setting the alignment length factor α in the range of 0.3–0.8, which has little impact on the performance. However, if we need to get more accurate SINE seeds, a larger copy number factor τ should be set. On the contrary, if we need high sensitivity, a smaller copy number factor is preferred. We plotted the change of the evaluation metrics with the change of the two factors in Figure  3.

### Cases of position-specific copy number profiles

**Figure 4:**
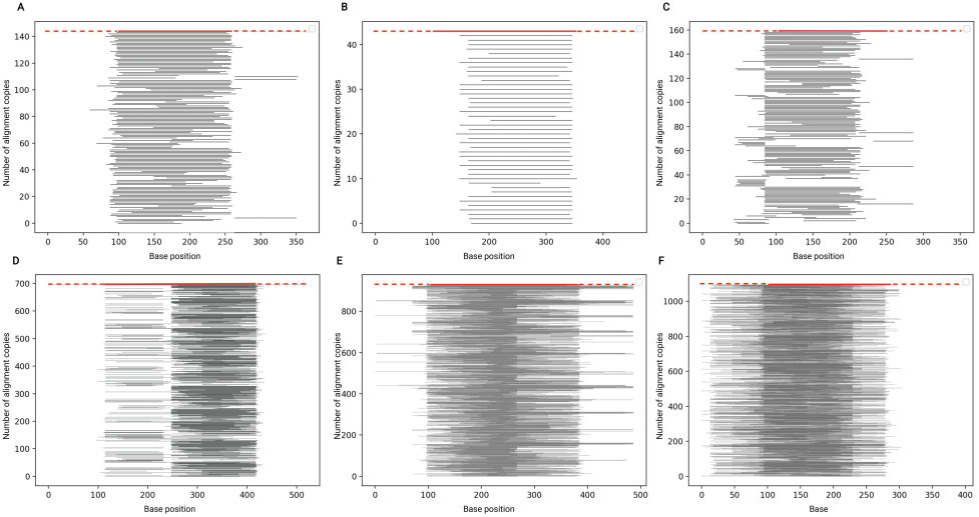
Examples of representative alignments of SINE copies. Red solid line: the output of HMMER. Red dashed line: the flanking region. Black/gray lines: the output of the alignment by BLAST. A)–C represent true SINEs, while (D)–(F) are profiles of non-SINEs. Step 3 in our tool can distinguish them.

The alignment between the repeat copies demonstrates different types of profiles, with examples of the representative ones shown in Figure  4. Among them, A–C are SINEs, while D–F are non-SINEs. In Figure  4A, a majority of the repeat copies are longer than the homologous sequence identified by HMMER. The extension, in this case, is very useful in generating more accurate SINE boundaries. In Figure  4B, a majority of the repeat copies are shorter than the sequence identified by HMMER. Adopting the repeat number threshold τ can easily remove the ending parts that are not part of the real SINE. In Figure  4C, some short, simple repeats can originate from the extended flanking regions. As these short repeats are not copied and pasted together with the SINE candidate, they are not part of the SINE and are removed from the sequence of this particular SINE.

Two truncated candidates belonging to other TEs are connected together as shown in Figure  4. In Figure  4D, both truncated parts are *Helitron*s. The candidate in Figure  4E is also removed from further analysis because the alignment is long and exceeds the typical length of SINEs. The candidate in Figure  4F is removed because of the shifted alignment. This candidate actually belongs to *Helitron*, and a large number of repeats are aligned in both flanking regions of the candidate sequence.

### The baseline performance of CNN-BiLSTM

In addition to screening the SINE candidates using multiple alignments and the presence of TSDs, we also attempted to filter the candidates through a machine learning approach. The performance of Convolutional Neural Network-Bidirectional Long Short-Term Memory (CNN-BiLSTM) in *A.* *thaliana* and rice is shown in Table  1. Its performance is comparable to that of customized screening (see “Materials and methods”) in *A.* *thaliana*, but slightly worse in rice. As deep learning benefits from large training data, the performance of deep learning-based methods may perform better with the increased availability of SINEs. By default, *AnnoSINE* uses customized screening in Step 3.

**Table 1:** Seed-level performance comparison of customized screening and deep learning-based methods

Model	TP	FP	FN
*A. thaliana*			
CNN-BiLSTM	251	0	14
Customized screening	251	0	14
Rice			
CNN-BiLSTM	219	8	16
Customized screening	227	0	12

### Benchmark experiments

**Figure 5:**
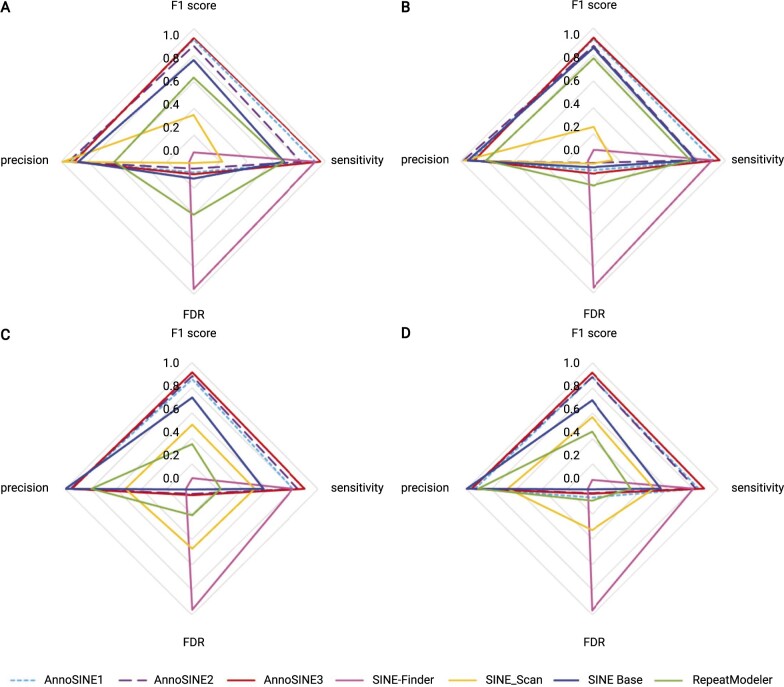
Performance comparison of different SINE annotation tools. A, Base-level evaluation in *A. thaliana*. B, Element-level evaluation in *A. thaliana*. C, Base-level evaluation in rice. D, Element-level evaluation in rice.

Our method is compared with four tools that can output SINE annotations, including SINE-Finder, SINE Scan, SINE Base, and RepeatModeler. The performance comparison is shown in Figure  5. The corresponding values are listed in Tables  2 and 3. The actual numbers of TP, FP, and FN at the element level and seed level are provided in the Supplemental Tables S2 and S3, respectively. SINE-Finder is a structure-based tool, which is capable of identifying intact SINEs with structural features. Once we obtain the complete SINE output by SINE-Finder, we annotate all of the SINE elements in the input genome with RepeatMasker (Chen, 2004). SINE Scan is also a structure-based tool as an enhanced version of SINE-Finder. It further verifies each complete SINE with copy number and other SINE features. The output SINE library from SINE Scan is used to annotate all SINEs in the genome using RepeatMasker as well. SINE Base is a database containing 234 known consensus sequences of SINE families, including those from *A.* *thaliana* and rice. RepeatModeler can output the SINE consensus sequences by identifying repetitive sequences and conducting TE classification. We thus directly use the consensus SINE sequences to annotate all SINEs in the genome via RepeatMasker.

**Table 2:** Element-level performance of different SINE annotation tools (optimal values are in bold for each evaluation metric)

Tool		*AnnoSINE1*	*AnnoSINE2*	*AnnoSINE*	SINE-Finder	SINE_Scan	SINE Base	RepeatModeler
*A. thaliana*	F1 score	0.918	0.868	**0.928**	0.081	0.255	0.851	0.772
	Sensitivity	0.910	0.780	**0.955**	0.901	0.146	0.772	0.734
	FDR	0.074	**0.020**	0.097	0.958	0.024	0.052	0.186
	Precision	0.926	**0.980**	0.903	0.042	0.976	0.948	0.841
Rice	F1 score	0.887	0.889	**0.924**	0.072	0.569	0.705	0.457
	Sensitivity	0.845	0.825	**0.890**	0.803	0.492	0.545	0.305
	FDR	0.067	0.035	0.040	0.963	0.327	**0.002**	0.092
	Precision	0.933	0.965	0.960	0.037	0.673	**0.998**	0.908

**Table 3:** Seed-level performance of different SINE annotation tools (optimal values are in bold for each evaluation metric)

Tool		*AnnoSINE1*	*AnnoSINE2*	*AnnoSINE*	SINE-Finder	SINE_Scan	SINE Base	RepeatModeler
*A. thaliana*	F1 score	**0.973**	0.805	0.971	0.498	0.531	0.695	0.522
	Sensitivity	**0.947**	0.673	0.943	0.730	0.361	0.532	0.327
	FDR	**0.000**	**0.000**	**0.000**	0.622	**0.000**	**0.000**	**0.000**
	Precision	**1.000**	**1.000**	**1.000**	0.378	**1.000**	**1.000**	**1.000**
Rice	F1 score	0.916	0.940	**0.974**	0.278	0.706	0.717	0.929
	Sensitivity	0.845	0.886	0.950	**0.967**	0.548	0.561	0.874
	FDR	**0.000**	**0.000**	**0.000**	0.837	0.008	0.007	0.009
	Precision	**1.000**	**1.000**	**1.000**	0.163	0.992	0.993	0.991

Below we summarize the performance of different tools.

- SINE-Finder has high sensitivity (>90% in *A.* *thaliana* and 80% in rice). However, it also has a high false discovery rate (FDR; ˃95%).
- SINE Scan reduced the FDR substantially (by 95% in *A.* *thaliana* and 45%-65% in rice) at the cost of sensitivity.
- SINE Base improved the tradeoff between sensitivity and precision. Both the base level and element-level accuracy can reach 99% for rice. The base level and element-level accuracies are ∼87% and 94% for *A.* *thaliana*. Nevertheless, the sensitivity is still unsatisfactory (50-60%).
- The performance of RepeatModeler is slightly worse than SINE Base in *A.* *thaliana*. In addition, its sensitivity is ∼35% lower than SINE Base in rice.
- In comparison, *AnnoSINE1* not only has the highest sensitivity at the base level and element level but also has better precision (˃90%) for *A.* *thaliana*. *AnnoSINE2* also has good performance. Although its sensitivity is 10% lower than SINE-Finder, its precision is 91% higher than SINE-Finder in *A.* *thaliana*. For rice, both *AnnoSINE1* and *AnnoSINE2* have the best sensitivity (˃95%) and precision (80-90%) at the base level, indicating that our tool can generate more accurate SINE boundaries. *AnnoSINE*, which integrates *AnnoSINE1* and *AnnoSINE2*, has the highest sensitivity and the best F1 score in all experiments.

#### Seed-level performance 

We quantify the seed-level performance by examining the overlaps between the standard library and the library generated via other tools. The performance comparison is shown in Table  3. If a seed sequence can be masked by the standard library with the minimum Smith–Waterman (SW) score of 225 and the minimum coverage of 50%, the two seeds are considered to have overlaps. Our pipeline *AnnoSINE* and SINE Scan are not associated with any FP at the seed level, suggesting the high precision of the two programs. However, when we apply RepeatMasker for annotating SINEs at the genome-scale, a small portion (3%–5%) of apparent FPs are generated using the seed sequences as the library. It is likely due to the fact that the seed sequences in the standard library and the test library are not exactly the same, causing a low level of discrepancy in the final annotation output.

It is worth noting that although RepeatModeler has high sensitivity (87%) and precision (99%) at the seed level for rice, its accuracy and sensitivity dropped substantially at the genome scale (sensitivity <30%). The decreased performance is mainly caused by two problems. First, the RepeatModeler’s seed sequences contain two consensus sequences over 1 kb in length. Although only a small region of the two long sequences is similar to SINE, the entire consensus sequence is annotated as a SINE sequence by RepeatModeler. Thus, the non-SINE portions of the consensus sequences lead to some FP annotations in the genome. In addition, there are 30 seeds (in the standard library) that are missed by RepeatModeler. Because those 30 seeds represent the abundant SINE families in the genome, many FNs are generated during the genome-wide annotation. As a result, the sensitivity is ˂30% at the genome-wide level.

#### Running time comparison

We also benchmarked the efficiency of SINE annotation tools on the MacBook Air platform with a 1.6 GHz Intel Core i5 processor and an 8 GB 2133 MHz LPDDR3 memory. Table  4 lists the running time, which refers to the period starting from the program taking the inputs until the completion of the SINE annotation. In other words, it includes all eight steps in Figure  2 for *AnnoSINE*. As expected, the running time depends on the genome size and proportion of SINE elements in the genome. Our pipeline can achieve good performance with reasonable running time.

**Table 4:** Running time (min) of different SINE annotation tools

Tool	*AnnoSINE1*	*AnnoSINE2*	*AnnoSINE*	SINE-Finder	SINE_Scan	SINE Base	RepeatModeler
*A. thaliana*	13	16	28	7	44	3	2,771
120.6 MB							
Rice	167	125	295	12	760	14	12,260
382 MB							

## Discussion

SINEs are short and highly heterogeneous, making SINE annotation more challenging than annotating other TEs. Although there are existing tools for annotating SINEs, they have various limitations. For example, SINE-finder has a very high false-positive rate (˃90%), whereas SINE Scan has very low sensitivity. SINE Base is entirely dependent on the known SINE library to implement homologous sequence identification, and it is unlikely to perform well for novel SINEs that are not contained in the known library. The current version leads to low sensitivity in rice. To generate more accurate SINE annotations, we developed *AnnoSINE*, which integrates all known features of SINEs for boosting SINE annotation. First, we apply the HMM for homology-based search to increase sensitivity. Then we compliment the homology-based approach with a structure-based search to maximize the pool of SINE candidates. Most importantly, we carefully examine and categorize the types of false positives within the SINE candidates and design corresponding filters for those false positives, which allows our tool to achieve both high sensitivity and precision.

Although *AnnoSINE* has the best performance among all tested tools, there is still room to optimize its performance. With more and more SINEs available, one future direction is to apply deep learning-based methods for SINE annotation. But this method requires a large amount of training data to prevent overfitting. In addition, deep learning models are not as accurate as pHMM-based models in identifying accurate loci of SINEs in genomes. In this work, we made several attempts to use deep learning models for SINE annotation. First, we implemented three well-studied deep learning models to distinguish SINEs from non-SINEs using the position-specific copy number profiles. Convolutional neural network-bidirectional long short-term memory (CNN-BiLSTM) has the same performance as the customized screening method in *A.* *thaliana*, while its performance is slightly worse in rice. As there are many parameters to train for the deep learning models, we suspect that inferior performance is mainly attributed to the limited training data. Second, we also formulated the SINE annotation problem as a learning problem using BiLSTM. In particular, the learning model can label each base as “inside SINE” or “outside SINE” and thus can use the output labels directly for genome-scale annotation. However, the final performance is much worse than the boundaries obtained by pHMM-based homology search. Thus, although an automatic pipeline based on learning can save users from adjusting the parameters, the limited training data at the current stage does not support its utility for this application. With the increased availability of sequenced genomes and their SINE annotations, deep learning-based methods may obtain some unique advantages in not only SINE annotation, but general TE annotation.

## Conclusions

SINEs are a group of highly diverse TEs with degenerate sequence and structural characteristics, making genome-scale SINE annotation difficult. In order to deliver better SINE annotation for plant genomes, we developed a SINE annotation tool, *AnnoSINE*, that takes genomic sequences as input and outputs a nonredundant SINE library, redundant library, and genome-scale SINE annotation. The benchmark experiments against several other existing tools show that *AnnoSINE* demonstrates a substantially better tradeoff between sensitivity and precision at both the base- and element-level evaluations. As the current SINE annotations in most plant genomes are far from ideal, we hope that this tool can make contributions in providing fast and accurate SINE annotations for more plant genomes in different taxa groups.

## Materials and methods

### Step 1: generating SINE candidates

Our tool *AnnoSINE* identifies SINE candidates using two methods in the first step. A homology-based search is utilized to find SINE candidates if the query genome is closely related to the genomes with known SINE annotations. If the query genome is distant from those genomes, structure-based de novo SINE search programs can be applied to identify putative SINE elements. To distinguish the two pipelines, we name them *AnnoSINE1* and *AnnoSINE2*, which represent homology- and structure-based methods for SINE candidate generation, respectively.

#### *AnnoSINE1*: homology search

With the increased availability of SINE annotation covering different plant taxa, the homology-based search would generate sensitive SINE identification in related plants, particularly those in the same family.

**Figure 6:**
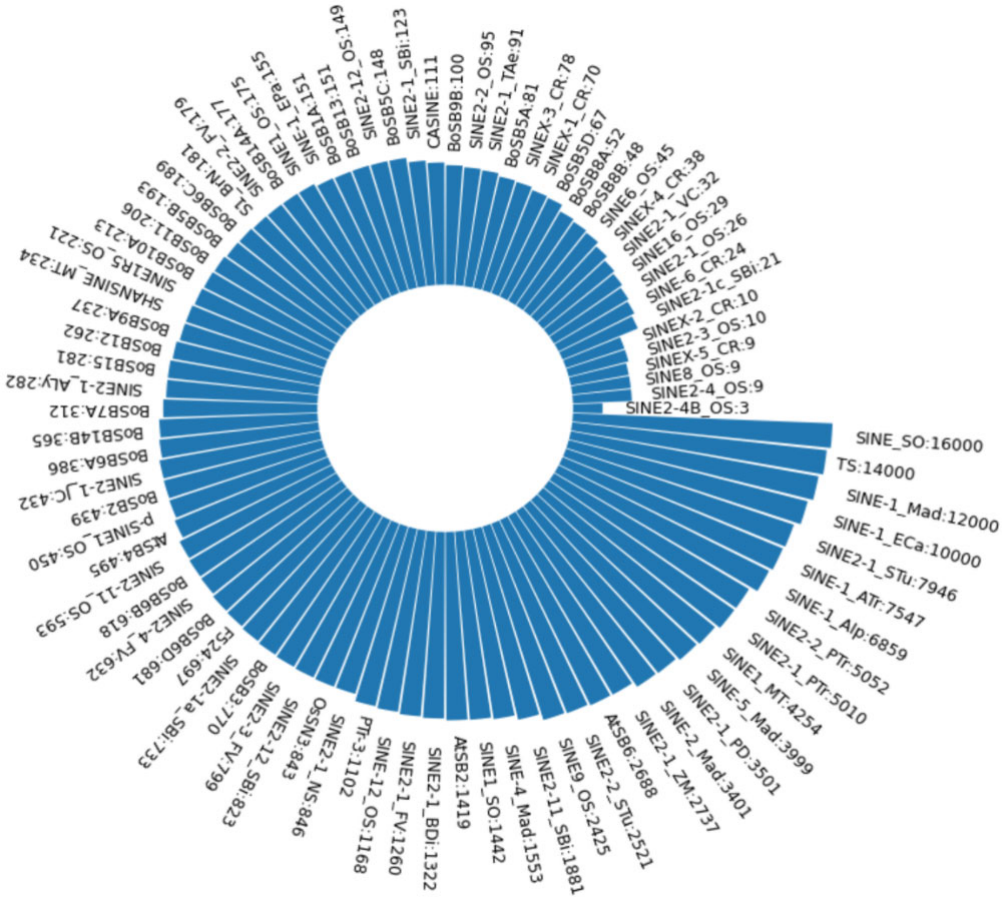
Number of sequences for each SINE family. A total of 88 pHMMs are constructed from the 88 families.

We apply profile HMM (pHMM)-based homology search because it is more sensitive in finding remote homologs than pairwise sequence alignment tools such as BLAST (Eddy, 2011). We first examined whether we can use the DFAM database (Hubley et al., 2016), which hosts numerous pHMMs for TE families, as the training data for SINE finding. We extracted 6,585 pHMMs from DFAM on the condition that the TE type of each pHMM is SINE and the model length is within 5,000. However, all of the extracted SINE pHMMs are from species in Animalia. Therefore, we cannot directly use DFAM and need to construct pHMMs with SINEs in plants. We extracted all the SINE sequences from training genomes and clustered them into the corresponding SINE families using the given annotation (family labels). There are 88 families in total, and their sizes are shown in Figure  6. Within each family, we conducted MSA using Clustal Omega (Sievers et al., 2011). Then the MSA is encoded into a pHMM via hmmbuild in HMMER3 (Eddy, 2008).

Once the pHMMs are built for all the families, our tool conducts a homology search for any query genome or assembled sequence using nhmmer in HMMER3. The default E-value is 1.0, but the users can adjust the threshold for a more stringent or lenient homology search.

#### *AnnoSINE2*: de novo SINE search using structural features

In order to identify potential SINEs that lack substantial sequence similarities with known SINEs, we use the structure-based de novo SINE search tool to generate the candidates. We chose SINE-Finder, the most sensitive de novo SINE search tool for identifying putative SINE sequences using structural-based features (Wenke et al., 2011). Although SINE-Finder may return many false candidates, they will be excluded in the following screening steps. SINE-Finder takes the whole genome as input. It slices the input genome into fragments of 10 kb with a 2 kb overlap, which is long enough for identifying complete SINEs located at the boundary of the fragments. It identifies the putative SINE candidates inside each fragment that has typical SINE molecular structural features. In particular, SINE-Finder identifies two motifs, boxes A ([GA][CGA]TGG) and B (CTTA[AG]A), in the tRNA-related region. It also examines the distance between A and B and the distance between B and the tail. The number of nts between boxes A and B ranges from 25 to 50, and the distance of box B and the tail is between 20 and 500 nts. In general, the tail region of SINEs consists of poly(A), poly(T), or other low complexity repeat sequences. The 5′-end TSD is located within 40-nt upstream of box A, and the 3′-end TSD is immediately adjacent to the 3′-end.

### Step 2: identification of TSD

Step 1 can produce a large number of SINE candidates. However, both homology search and de novo structure-based search may return false candidates. To further screen for bona fide SINEs, we examine TSDs around each candidate SINE, which is an essential feature of SINEs (Vassetzky and Kramerov, 2013). However, as TSD is short and does not always exist immediately adjacent to the apparent termini of each SINE, the search region of TSD needs to be carefully determined. First, as the length of the SINE tail can vary substantially, the detection range on the 3′-terminal should be longer than the 5′-terminal (Vassetzky and Kramerov, 2013). Second, considering that the length of TSD is generally from 10 to 20 nt, we extend 30-nt upstream of the 5′-terminal and 50-nt downstream of the 3′-terminal by default. We search for the TSD in the flanking regions and exclude the candidates without TSDs. This step is very effective in eliminating false positives. There are also cases where TSDs cannot be detected owing to the mutation or insertion/deletion after insertion. These SINEs will be missed in this step. However, as long as one intact SINE with TSD (a potential seed sequence) from the same family is detected, we will be able to annotate and output the missed ones in the final step (Step 8) of our pipeline.

### Step 3: improving SINE annotation by examining the alignment of SINE copies

**Figure 7:**
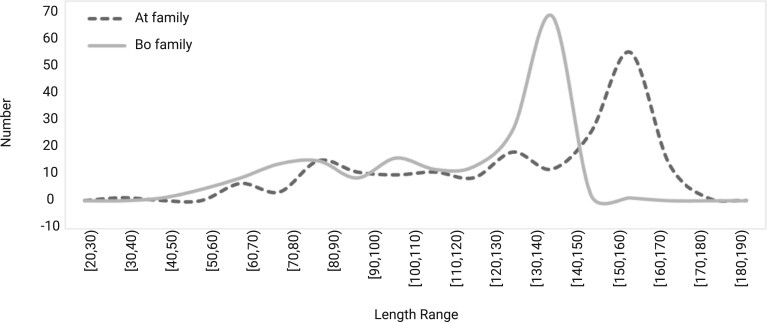
The SINE length histogram in Arabidopsis and Brassica. Black dashed line: Arabidopsis family. Gray solid line: Brassica family.

TSD-based screening cannot remove all false candidates. In addition, homology or structure-based tools may not yield accurate SINE boundaries in the genome. For example, HMM could be constructed from SINEs that are shorter than the SINEs in the input genome. Thus, homology search can underestimate the length of actual SINEs. For example, although Arabidopsis and Brassica share many SINEs with decent sequence similarities, their length distribution has lineage-specific patterns. The SINEs of Arabidopsis tend to be longer than their homologs in Brassica, as shown by the length distribution profiles of SINEs in Figure  7. To overcome this problem, we extend the initial SINE annotation by Step 1 to accommodate possibly longer SINEs.

**Figure 8:**
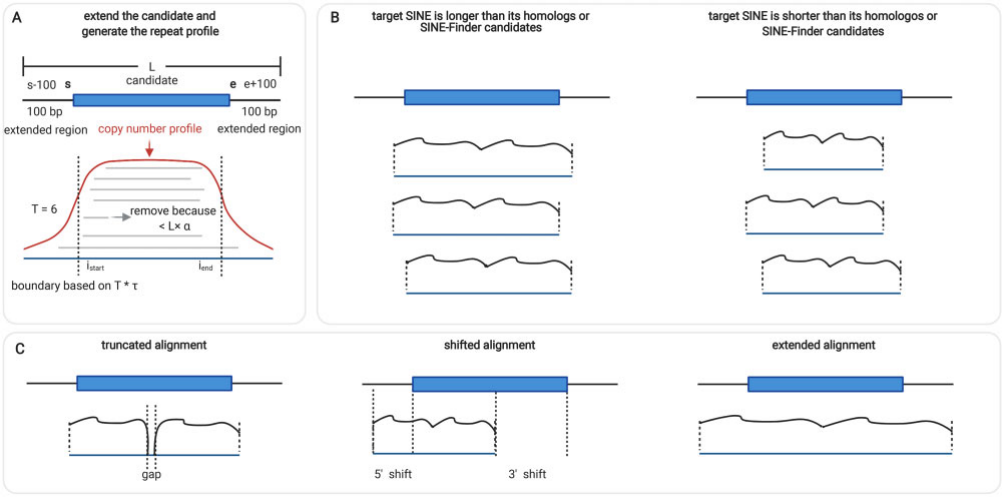
Repeat profiles. A, Copy number profile generation. α: length factor. τ: copy number factor. B, Cases where the target SINEs are longer or shorter than their homologs in the reference dataset. C, Truncated, shifted, and extended profiles, which usually represent non-SINEs.

Specifically, we extend both 5′- and 3′-ends of each SINE candidate for 100 bp, where the 5′- and 3′-ends are determined by Step 1. As shown in Figure  8A, let the starting and ending positions of the SINE element be s and e. After extension, the new search region is extended to s-100 and e+100. Then the region between s-100 and e+100 is used as a query for BLAST to search for its copies in the whole genome with an e-value cutoff 1e−10. As BLAST is a local alignment tool, some short alignments that are not real SINE copies will be returned. In order to remove non-SINEs that fortuitously share local sequence similarities with the SINE pHMMs, we only keep the alignments with a length above L×α, where L=|e-s+201| and α is the length factor that indicates the required percentage of alignment length versus L. By default, α=0.3.

#### Refining the SINE boundaries

All the returned SINE copies from BLAST search are used for producing an MSA, which is constructed using the query as the template. The MSA will be converted into a position-specific copy number profile, where each position contains the number of aligned bases. Considering that the SINE copies should be highly similar, we refine the SINE boundaries using the copy number profile.

Let the number of sequences that can be aligned with the candidate SINE be T. Let a locus between s-100 to e+100 be i. The first base $i_{\mathrm{start}}$ on the 5′-end and the last base $i_{\mathrm{end}}$ on the 3′-end with copy numbers above τ×T are used as the new boundaries of the candidate SINE, where copy number factor τ indicates the required percentage of copies for a SINE. Then, we examine the position-specific copy number profile to further distinguish SINEs from other elements.

#### Exclusion of non-SINEs through position-specific copy number profiles

We developed two methods to distinguish SINEs from non-SINEs using the position-specific copy number profiles. As shown in Figure  8C, non-SINEs tend to have truncated, shifted, or very long repeats beyond the original candidate boundaries. Thus, in the first method, we employ empirical parameters to screen these, such as the boundary shift cutoff and gap size. The advantage of this method is that the parameters can be interpreted and understood by users. However, the cutoffs may need to be adjusted for different inputs in order to achieve optimal performance. In the second method, we employ machine learning models to automatically learn the patterns in the copy number profiles in SINEs and apply the models to detect non-SINEs in the framework of binary classification. The advantage of this method is the automatic feature learning, which does not require customized cutoffs from users.

#### Method 1: customized screening

We observed three types of repeat family structure profiles that are not associated with true SINEs. We sketched them in Figure  8C. The first is a truncated alignment, which usually belongs to two different TEs joined together by mistake or nested insertion rather than a single SINE, for example, two *Helitron*s. We can identify it by the presence of a gap in the alignment, which corresponds to positions with repeat number <τ×T. Specifically, any candidates with a gap over ten bp will be removed. The second is a shifted alignment far away from the original boundaries s and e. A majority of these profiles belong to other TEs, including miniature inverted-repeat TEs (MITEs), Mutator-like TEs, and Helitrons. We define the shift as $\max\left(|\left|i_{\mathrm{start}}-s\right||,\;\left|{|i}_{\mathrm{end}}-\mathrm{e|}\right|\right)$ and remove the ones with the shift value >50 nt. Third, there are some extremely long alignments extending beyond s-100 or e+100. These extended profiles are most likely part of a long repeat sequence such as LINEs rather than SINEs. By default, we discard such elements.

#### Method 2: learning-based screening

**Figure 9:**
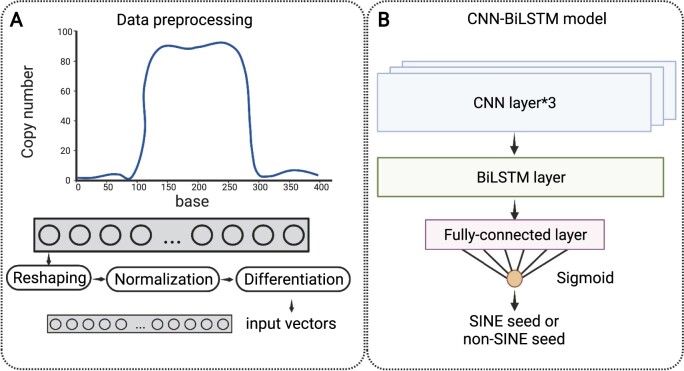
Architecture of the CNN-BiLSTM deep learning model. A, Generating the input vectors for CNN-BiLSTM. B, The CNN-BiLSTM model.

In contrast to method 1, which uses customized parameters to distinguish the copy number profiles of SINEs and non-SINEs, method 2 uses a learning-based automatic pipeline for the classification of SINEs. By using the position-specific copy number profiles of SINEs and non-SINEs as the positive and negative training data, we implemented three binary classification models: CNN, BiLSTM, and CNN-BiLSTM. Of the three models, the hybrid CNN-BiLSTM has the best performance in distinguishing SINEs from non-SINEs as shown in Supplemental Table S1. Figure  9 shows the architecture of our CNN-BiLSTM. The architectures of CNN and BiLSTM are shown in Supplemental Figure S1. It can retain the dependencies of the historical position-specific copy number information in two directions based on the extracted local patterns of the profile. In the Supplementary document, we provide the details about the training data, the pre-processing steps, and the model architecture.

### Step 4: SINE superfamily classification

After we annotate SINEs, we classify them into superfamilies by examining the origin of SINE heads (Vassetzky and Kramerov, 2013). At the 5′-terminal, there is a region related to cellular RNA synthesized by RNA polymerase III, including tRNA, 7SL RNA, and 5S rRNA (Mao and Wang, 2017). Superfamilies can be determined by scanning each candidate sequence against the libraries of three types of RNA. However, when the candidate sequences are nearly identical with these RNAs in Rfam (Kalvari et al., 2021; E-value = 1e-15 by default), it is possible that they are part of these noncoding RNAs rather than SINEs. Therefore, these candidate sequences are also excluded from the final output.

### Step 5: tandem repeat

Tandem repeats in DNA are duplicated or similar copies of two or more continuous nt patterns (Benson, 1999). SINEs may contain a small proportion of the tandem repeats. If the majority of sequences are tandem repeats, they are more likely part of the low-complexity sequences. We employ the tandem repeat finder (Benson, 1999) to eliminate the candidates with a substantial fraction of tandem repeats (70% in length).

### Step 6: terminal inverted repeat

MITEs are prevalent in plants (Feschotte et al., 2002). With similar size to SINEs, they constitute a fraction of false-positive SINEs. MITEs are flanked by the terminal inverted repeats (TIRs) that are 10 nt or longer, whereas SINEs do not have TIRs. Thus, we utilize the feature to further verify the candidate SINEs. Specifically, we search for TIRs using the inverted repeat finder (IRF; Warburton et al., 2004) in two areas: 1) between 5′-terminal TSD end position $i_{\mathrm{start}}$ and $i_{\mathrm{start}}+50$; 2) between $i_{\mathrm{end}}-50$ to 3′-terminal TSD start position $i_{\mathrm{end}}$. Candidate elements containing TIRs are excluded from the output.

### Step 7: nonredundant library

The nonredundant library is generated by searching and removing the redundant seed sequences. We applied CD-HIT with the identity cutoff of 80% to group similar SINEs into one cluster (Ou and Jiang, 2018). For each cluster, the longest one is selected as the representative seed of this cluster.

### Step 8: genome-wide SINE annotation

After constructing the seed library, we use RepeatMasker (https://www.repeatmasker.org/; Chen, 2004) for high-quality SINE element annotation. We only identify homologous sequences with up to 40% divergence from the intact SINEs in the library built in Step 7. NCBI BLAST is selected as the search engine. “-q” search mode is used to ensure quick and sensitive annotation.

### Experiment datasets

In order to build the pHMMs in Step 1, we need to retrieve some known SINEs as the training data for model construction. There are many plant genomes with TE annotations at NCBI’s Genbank (https://ftp.ncbi.nlm.nih.gov/genomes/genbank/plant/). We downloaded all plant genome annotation files in the general feature format that contain SINE annotation and used them as our training data. Of the 62 plant genomes with SINE annotations, 60 are from flowering plants. They cover multiple families, including Amborellaceae, Brassicaceae, Fabaceae, Solanaceae, Myrtaceae, Rosaceae, Euphorbiaceae, Arecaceae, Salicaceae, and Poaceae. In order to test the performance of SINE annotation on “new” species, we conduct cross-species experiments by removing the test species from the training data. Thus, there is no overlap in the SINE sequences between the training (for pHMM construction) and testing datasets.

### Standard SINE library

Specifically, we choose *A.* *thaliana* and rice as the test species because we can obtain their SINE annotations with relatively high confidence.

We obtained the TE annotation of *A.* *thaliana* from NCBI’s Genbank, which annotated TEs in the whole genome based on the repeat library from Repbase. We then derived the standard nonredundant SINE seed library from the SINE annotation using CD-HIT with an 80% identity cutoff.

The manually curated rice SINE standard library was constructed based on a rice TE library from a previous study (Ou et al., 2019). All the SINE sequences in the previous library were re-examined and compared with other TEs in the library for potential errors or mis-annotations. Briefly, for each SINE sequence in the library, multiple family members of each SINE, as well as 100-bp flanking sequences on each side, were retrieved, and an MSA (see Step 3) was generated. The MSA was manually examined for the position of boundary and presence of TSD. For a few elements, the 5′-termini for some members could extend much further than the sequences in the library, and the extended portion was similar to known LINEs. As a result, those elements were likely truncated versions of LINEs and were excluded from the library. To identify SINE elements that were missed in the previous study, two approaches were employed. First, the initial false-positive elements from *AnnoSINE* were examined as above. While most false positives were truly false positives, three represented true SINE elements and were added to the standard library. Second, the rice genomic sequences were masked using known TEs. The repetitive sequences in the unmasked portion of the genome were collected using RepeatModeler (https://www.repeatmasker.org/), and sequences that are shorter than 800 bp were examined. A few new SINE families were identified and added to the standard library. To build a more comprehensive library, all the related intact SINE elements in the genome (<5 bp truncated at each end), with ˃20% divergence (based on RepeatMasker’s output) to the element in the library were collected, and redundancy was reduced as described in Step 7. Thereafter, the sequences of nonredundant distant relatives were added to the library to form the final standard library for subsequent analysis (Supplemental Files S1 and S2).

### Evaluation metrics

**Figure 10:**
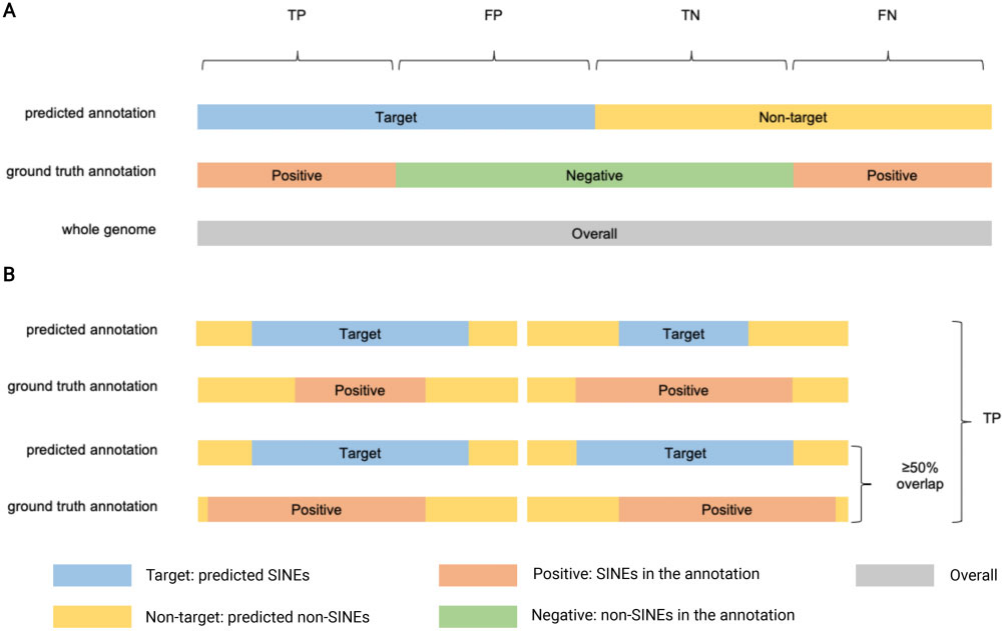
The evaluation metrics and their definitions. A, base-level evaluation metrics. B, element-level evaluation metrics.

As shown in Figure  10, the whole genome is categorized into four parts compared to the annotation using the standard library, which is true positive (TP; SINEs are identified), false negative (FN; SINEs are not identified), false positive (FP; non-SINEs are identified as SINEs), and true negative (TN; non-SINEs are not identified as SINEs). We present two sets of evaluation metrics, which focus on base-level and element-level evaluation, respectively. They have different sets of ground truth, with the former using all bases in SINEs as the positive set while the latter using the SINE sequences as the positive set. The base-level evaluation compares the prediction against the known annotation for each base. The element-level evaluation focuses on examining whether each known SINE has a sufficient overlap (˃50%) with a prediction. Given these definitions, we have the following equations. Some previous works also presented specificity, which is TN/(FP + TN). However, as the fraction of SINE is very small (so is the FP) compared to the entire genome, the value of FP is usually several orders of magnitude smaller than TN at the base level. In this case, specificity is always high (close to 1.0) regardless of the performance of the tools; we, therefore, will not report specificity here.

(1) $$\text{Sensitivity}=\frac{\text{TP}}{\text{TP}+\text{FN}}$$

(2) $$\text{Precision}=\frac{\text{TP}}{\text{TP}+\text{FP}}$$

(3) $$\text{False}\;\text{discovery}\;\text{rate}=\frac{\text{FP}}{\text{TP}+\text{FP}}$$

(4) $$F1\;\text{score}=\frac{2TP}{2\text{TP}+\text{FP}+\text{FN}}$$

We also evaluate the performance of SINE annotation at the seed level in *A.* *thaliana* and rice. In order to compare our seed sequences against the standard TE library, we use the RMBlast search engine in RepeatMasker with an SW score of 225 according to the parameter settings in (Ou et al., 2019). In addition, we also require at least 50% coverage of the query sequence.

## Supplemental data 

The following materials are available in the online version of this article.

**Supplemental methods.**

**Supplemental Figure S1.** Architectures of CNN and BiLSTM deep learning models. (A) CNN. (B) BiLSTM.

**Supplemental Table S1.** Performance comparison of learning-based methods.

**Supplemental Table S2.** The values of TP, FP, and FN of different SINE annotation tools at the element level (see Methods about the definition of element level).

**Supplemental Table S3.** The values of TP, FP, and FP of different SINE annotation tools for seed level evaluation.

**Supplemental File S1.** Standard SINE library for rice.

**Supplemental File S2.** Standard SINE library for *A.* *thaliana*.

## Supplementary Material

kiab524_Supplementary_Data

## Notes

Y.L. and Y.S. conceived the SINE annotation project and designed the experiments; N.J. provided guidance on screening SINE candidates and curated the standard *Oryza sativa* SINE library used in the experiment. Y.L. conducted the experiments and analyzed the results under the guidance of Y.S. and N.J. Y.S., N.J., and Y.L. wrote and approved the manuscript.

The author responsible for distribution of materials integral to the findings presented in this article in accordance with the policy described in the Instructions for Authors (https://academic.oup.com/plphys/pages/general-instructions) is Dr Yanni Sun (yannisun@cityu.edu.hk).
