# Supplementary material for: AnnoSINE: a short interspersed nuclear elements annotation tool for plant genomes
Source: Plant Physiol. 2021 Nov 18;188(2):955–70. doi: 10.1093/plphys/kiab524 (PMC8825457; doi:10.1093/plphys/kiab524)
Supplement: kiab524_Supplementary_Data [file kiab524_supplementary_data.pdf]

***AnnoSINE*: A new SINE annotation tool for plant genomes**

Yang Li, Ning Jiang, and Yanni Sun

SUPPLEMENTAL METHODS

Method 2: learning-based screening

Besides the default method that uses customized parameters to distinguish the position-specific copy number profiles of SINEs and non-SINEs, we also implemented three deep-learning models in order to conduct automatic classification. The three models are Convolutional Neural Network (CNN), Bidirectional Long Short-Term Memory (BiLSTM), and their hybrid model CNN-BiLSTM. The basic architectures of CNN and BiLSTM are sketched in Supplemental Figure S1.

In order to train the learning models, we first need to construct the positive and negative training data, which are copy number profiles of SINEs and non-SINEs. To obtain the profiles, we process the training data following the same preceding steps (homology search or structural SINE search and TSD search). Then BLAST is applied to construct the MSA, from which we build the copy number profiles. Based on the available seed SINE annotations and repeat-Masker's outputs for the training genomes, we distinguish the copy number profiles into positive and negative training data. Finally, we keep 2,480 training elements to achieve balanced training data, with half as the positive and the other half as the negative. For the training data, we implement data reshaping, normalization, and differentiation in the preprocessing procedures. The test species are still *Arabidopsis thaliana* and rice (*Oryza sativa*).

Let the ith copy number profile, which is the ith input to the learning models, be a vector C^i. Then, the jth position in the vector is C^i_j. As CNN needs an input of fixed size, we thus use the maximum length of SINE, which is 1000 bp, as the fixed vector size. For C^i with a length smaller than 1000, we conduct padding by adding 0 from the end to 1000. Thus each vector can be expanded as $\{C^i_1, C^i_2, \ldots, C^i_{1000}\}$. Let the maximum copy number of different positions for C^i be $max(C^i)$. Then, we will normalize the data so that all variables are on the same scale. The normalized value for each position j is $\frac{C^i_j}{max(C^i)}$. Finally, we multiply $\{+1, -1\}$ to differentiate original SINE region $[s, e]$ and extended region from $[s-100, s)$ and $(e, e+100]$.

CNN applies multiple layers of convolution to learn the features from the copy number profiles. We choose three 1D convolutional layers with 32, 16, and 8 filters respectively. The kernel size is 3, and the stride length is 1. The activation function rectified linear unit (ReLU) is applied to increase the nonlinearity in the feature map. We also use an initializer that generates tensors with normal distribution and MaxNorm weight constraints during the training process. LSTM can incorporate the dependence between different positions, and thus it may help improve the classification accuracy. Accordingly, we also apply BiLSTM to combine the forward and backward LSTMs. Finally, we implement the third model (CNN-BiLSTM) by using the learned features through CNN as input to BiLSTM.

SUPPLEMENTAL FIGURE AND TABLES

Supplemental Figure S1. Architectures of CNN and BiLSTM deep learning models. A. CNN. B. BiLSTM.

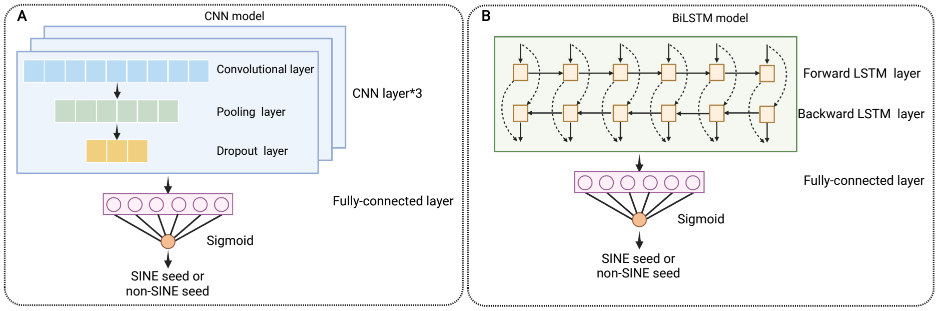

Supplemental Table S1. Performance comparison of learning-based methods.

Model	TP	FP	FN
CNN	251	0	14
BiLSTM	251	0	14
CNN-BiLSTM	251	0	14

(a) *Arabidopsis thaliana*

Model	TP	FP	FN
CNN	226	17	13
BiLSTM	224	34	15
CNN-BiLSTM	219	8	16

(b) rice

Supplemental Table S2. The values of TP, FP and FN of different SINE annotation tools at the element-level (see Methods about the definition of element-level).

Tool	TP	FP	FN
AnnoSINE1	503	40	50
AnnoSINE2	462	32	91
AnnoSINE3	528	57	25
SINE-Finder	498	11,262	55
SINE_Scan	81	2	472
SINE Base	454	25	134
RepeatModeler	406	93	147

(a) *Arabidopsis thaliana*

Tool	TP	FP	FN
AnnoSINE1	7,185	515	1,315
AnnoSINE2	7,010	252	1,490
AnnoSINE3	7,564	316	936
SINE-Finder	6,824	175,154	1,676
SINE_Scan	4,183	2,031	4,317
SINE Base	4,630	7	3,870
RepeatModeler	2,596	262	5,904

(b) rice

Supplemental Table S3. The values of TP, FP, and FP of different SINE annotation tools for seed-level evaluation.

Tool		*AnnoSINE1*	*AnnoSINE2*	*AnnoSINE3*	SINE-Finder	SINE_Scan	SINE Base	RepeatModeler
Arabidopsis thaliana	TP	249	206	248	172	91	131	86
	FP	0	0	0	317	0	0	1
	FN	14	57	15	91	172	132	177
rice	TP	202	203	227	231	131	134	209
	FP	0	0	0	1,189	1	1	2
	FN	37	26	12	8	108	105	30
